# Effects of motor fatigue on walking stability and variability during concurrent cognitive challenges

**DOI:** 10.1371/journal.pone.0201433

**Published:** 2018-07-26

**Authors:** Pei-Chun Kao, Michaela A. Pierro, Konstantina Booras

**Affiliations:** 1 Department of Physical Therapy, University of Massachusetts Lowell, Lowell, Massachusetts, United States of America; 2 Biomedical Engineering and Biotechnology Program, University of Massachusetts Lowell, Lowell, Massachusetts, United States of America; University of Illinois at Urbana-Champaign, UNITED STATES

## Abstract

Cognitive-motor interference, a negative influence on the performance of one or both tasks, is manifested when simultaneously performing a cognitive and a motor task. Motor fatigue reduces the ability of generating a required force level. However, little is known about the effects of motor fatigue on the cognitive-motor dual-tasking performance, an important capability during our daily lives. This study investigated how motor fatigue affects dual-task walking performance. Eighteen healthy younger adults walked on a treadmill under three different conditions: walking only, walking while receiving the Paced Auditory Serial Addition Test (PASAT) or a modified Stroop test before and after a lower-extremity fatiguing exercise. We computed dynamic margins of stability (MOS), step and joint kinematic variability, and short-term local divergence exponent (LDE) of the trunk motion. We found that subjects had similar values of short-term LDE during all conditions, indicating that local stability was not affected by the motor fatigue or dual-task conditions. Compared to the baseline, subjects had significantly greater mean MOS after the fatiguing exercise by walking with greater step length and width while having significantly greater gait variability. In contrast, subjects walked with similar mean MOS but significantly less gait variability during the dual-task conditions, indicating that subjects used different adaptive strategies when walking with motor fatigue and during dual-task conditions. There were no significant differences in the number of errors for the two cognitive tests before and after the fatiguing exercise. The current findings demonstrate that motor fatigue does not affect cognitive but motor performance in younger adults.

## Introduction

Motor fatigue has been shown to reduce neuromuscular performance such as a temporary decline in the ability of generating a desired force level [1]. Motor fatigue would also affect an individual’s precision in motor control due to the deteriorated proprioception (e.g., decreased joint position sense, force-matching ability, etc.), movement coordination and reaction time [2–6]. Regarding the effects of motor fatigue on gait, previous studies showed that healthy subjects used adaptive strategies to walk with a more cautious pattern by lowering walking speed, reducing step length and/or increasing step width [7]. The adaptive strategies might help maintain an individual’s walking stability by offsetting the effects of increased variability in motor performance caused by the leg muscle fatigue.

Cognitive-motor interference, a negative influence on the performance of one or both tasks, is manifested when simultaneously performing a cognitive task and a motor task [8]. It was suggested that the dual-task interference could be explained by two main theories: the limited attentional capacity sharing and the bottleneck models [9]. The limited attentional capacity sharing model assumes that the processing capacity of the brain is limited. Thus, a competition for attention or information processing resources during dual-tasking would lead to compromised performance of either or both tasks when the total attention required exceeds the capacity of an individual. The bottleneck model assumes that certain critical tasks must be carried out sequentially and thus, a bottleneck arises when the information from two different tasks are processed by similar neural networks. The effects of cognitive-motor interference have been found in multiple motor tasks such as a finger button pressing, standing, walking, complex visual-motor task (e.g., driving scenario), etc. [9–12].

Little is known about the effects of motor fatigue on the cognitive-motor interference such as how the compromised motor performance due to motor fatigue affects the cognitive-motor dual-tasking performance, an important capability in our daily lives. People like soldiers and firefighters would have a higher chance to reach motor fatigue compared to others because they carry heavy equipment and perform rigorous motor tasks that also demand a high degree of attention and information processing. When they reach motor fatigue, it is not clear if motor fatigue also affects their capacity of handling two tasks simultaneously. A reduction in the dual-tasking capability may increase more errors during the information processing or result in greater variability of motor performance that could potentially lead to physical injuries during the mission.

There are limited studies that investigated the effects of motor fatigue on the walking or cognitive performance under single or dual-task conditions. Granacher et al (2010) [13] found that, after muscle fatigue of knee extensors and flexors, healthy young adults significantly reduced walking speed and stride length during the walking only condition but did not alter those sagittal-plane gait parameters during the dual-task condition. In addition, they found that the performance of the cognitive task was even improved after the muscle fatigue. In Granacher et al (2010), a short testing duration (i.e., 10-meter overground walking) and a less challenging cognitive test (i.e., serial subtractions by three) were administered. The experimental setup of Granacher et al (2010) might not be able to reveal the effects of muscle fatigue on dual-task walking performance because the total attention required may not exceed the total information processing capacity of an individuals and thus, only led to a minimal negative influence on the gait.

The purpose of this study was to investigate how motor fatigue affects walking, cognitive and dual-task walking performance. To better reveal the effects of muscle fatigue on gait, we fatigued more major muscle groups responsible for walking than simply the knee muscles. In addition, we used two cognitive tests, paced auditory serial addition test (PASAT) [14] and Stroop test [15], which are considered quite challenging to most people. For the walking performance, specifically, we explored the effects of dual-tasking on subject’s gait variability, local dynamic stability and dynamic margins of stability. We hypothesized that healthy subjects would show reduced walking stability, increased gait variability and more errors in the cognitive tasks after reaching motor fatigue.

## Materials and method

### Subjects

Eighteen healthy young subjects without color blindness based on the self-report (10 females, 8 males, age: 19.8 ± 1.1 years, mean ± SD) gave written informed consent to participate in the study. This study complied with the Declaration of Helsinki and was approved by the Institutional Review Board of the University of Massachusetts Lowell (#17–078).

### Experimental protocol

All subjects walked on a treadmill at 2.8 mph (1.25 m/s) under three different conditions: walking only, walking while receiving the paced auditory serial addition test (PASAT) or a modified incongruent color-word Stroop test (Stroop) before and after a submaximal muscle fatiguing exercise session. Walking speed has been shown a confounding factor for gait variability [16]. To control walking speed during dual-tasking, we examined treadmill walking. During the PASAT, subjects were given a single digit number every two seconds through the auditory recording and asked to add each new number to the one immediately prior to it. During the Stroop test, subjects were visually presented an image consisting of the name of one of five colors, printed in text of a different color. These images were projected onto the wall serially and changed randomly every 1.25 seconds. To increase the difficulty of the Stroop test, subjects were instructed to identify the color of the text for those printed in a color other than red and read the name of the text for those printed in red. Subjects also received the cognitive tests in a seated position. Subjects were asked to provide their answers verbally for the two cognitive tests. Each trial was tested for 3 minutes and in a pseudo-randomized order. Prior to the testing, subjects were given time to familiarize themselves to the treadmill walking and the two cognitive tests.

The submaximal muscle fatiguing exercise session included leg presses, calf and toe raises. The exercise session required concentric and eccentric contractions of the Quadriceps, Hamstrings, Gluteus maximus, ankle plantarflexors and dorsiflexors. We first determined each subject’s one repetition maximum (1-RM) during the leg press task. Subjects then performed leg presses against the load equivalent to 80% of their 1-RM at the pace of 50 beats per minute (bpm) for 2 minutes or until voluntary exhaustion. To determine if subjects have reached motor fatigue of performing leg presses, subjects rested for 1 minute and then attempted to perform two consecutive leg presses at 85% of their 1-RM or 80% of 1-RM plus 20 lbs. If subjects were able to complete two consecutive leg presses at 85% of 1-RM or 80% of 1-RM plus 20 lbs, they would then perform another 2-minute bout of leg presses at 80% of 1-RM or until voluntary exhaustion. Subjects who could only perform one leg press or none at 85% of 1-RM or 80% of 1-RM plus 20 lbs were considered as having reached motor fatigue in performing leg presses and the leg press exercise would be terminated. Following the leg press task, subjects performed calf raises during standing at 90 bpm while holding a preferred dumbbell weight and performed toe raises against a Theraband in a seated position until voluntary exhaustion.

### Data acquisition and analysis

We collected three-dimensional (3-D) kinematics while subjects walked on the treadmill (RTM 600, Biodex Medical Systems, Shirley, NY, USA). The 3-D kinematic data were recorded using an 8-camera video system (100Hz, Motion Analysis Corporation, Santa Rosa, CA, USA) with reflective markers attached on the lower body, trunk and over the back of the neck (C7 vertebra). We used commercially available software (Visual3D, C-Motion Inc., Germantown, MD, USA) to perform initial data processing. Subject’s answers for the two cognitive tests were audio-recorded through a Bluetooth headset.

We quantified local stability by computing short-term local divergence exponents (LDE) of the trunk motion based on the reconstructed state spaces of C7 vertebral marker movement. The motions of C7 vertebral marker were used to represent trunk movement during walking. Procedures to compute short-term LDE were well established and stated in the previous studies [17–19]. For this study, we extracted data of 150 continuous strides for each trial and re-sampled the data to 15,000 total points, approximately 100 data points per stride. Delay embedded state spaces with an embedding dimension of 5 were reconstructed independently from the anterior-posterior (A-P), mediolateral (M-L), and vertical (VT) velocities of filtered C7 vertebral marker data, including the original data and its time delayed copies.

S(t)=[x(t),x(t+τ),x(t+2τ),x(t+3τ),x(t+4τ)], (1)

where *S*(*t*) was the 5-dimensional state vector, *x*(*t*) was the original 1-dimensional C7 vertebral marker velocity data, and *τ* was the time delay. We used fixed time delays of 25, 30 and 15 data samples for the A-P, M-L, and VT directions, respectively, for all trials. LDE quantified how quickly neighboring movement trajectories in a state space diverge over time. Briefly, we identified nearest neighbors and calculated Euclidean distances (i.e., divergence) between neighboring trajectories in the state space as a function of time. We averaged the logarithmic divergence over all original pairs of initially nearest neighbors.

$$y(i)=\frac{1}{\Delta t}\langle \ln\left[d_{j}(i)\right]\rangle ,$$ (2)

where *d*_*j*_(*i*)represents the Euclidean distance between the *j*^th^ pair of initially nearest neighbors after *i* discrete time steps (i.e., *iΔt*) and < > represents the average over all *j* pairs. Short-term LDE was estimated from the slope of a linear least-square fit to the mean log divergence curve across the span of 0–100 data samples [16, 20]. A positive, larger value of short-term LDE indicates more unstable and sensitive to local perturbations.

Dynamic margins of stability (MOS) was computed as the distances between the extrapolated center of mass (COM) positions (X_CO_M) and the boundaries of the base of support [21]. The X_CO_M position was calculated as:

$$X_{CO}M=COM+\frac{\dot{COM}}{\omega_{0}},$$ (3)

where COM and $\dot{COM}$ are the COM position and velocity of the whole body, respectively, and

$$\omega_{0}=\sqrt{\frac{g}{l}},$$ (4)

where *g* is the gravitational constant (9.81m/s^2^) and *l* is the equivalent pendulum length and taken as the height of the COM position during standing. We computed anterior-posterior MOS (MOS_AP_) as the A-P distance between the X_CO_M and the front toe marker of the leading foot. Mediolateral MOS (MOS_ML_) was computed as the lateral distance between the X_CO_M and the lateral toe marker of the leading foot. We will calculate MOS_AP_ and MOS_ML_ at heel strikes for each foot [22].

We computed the standard deviation of step width, length and time from each trial. We also calculated mean standard deviation (meanSD) of the lower-limb joint motions, C7 marker positions and velocities. The meanSD of the C7 marker positions and velocities quantify overall variability of subjects’ displacements (i.e., drift) on the treadmill and stride-to-stride trunk movement variability, respectively [16]. The meanSD of the lower-limb joint motions, trunk positions and velocities were calculated across strides at each normalized time point (0–100%) of the gait cycle and then averaged over the whole gait cycle to produce a single measure of the mean variability for each trial.

### Statistics

We first transformed the non-normally distributed data into a normal shape using the Box-Cox transformation [23] in MatLab (MathWorks, Inc., Natick, MA, USA). We then used repeated measures ANOVAs to test for differences in the stability and variability measures between the two time periods (before and after the fatiguing exercise session, PRE and POST) and three walking conditions (walk only, PASAT, and Stroop). We also used separate repeated measures ANOVAs to test for differences in the accuracy of the two cognitive tests (the number of errors) between the two time periods (PRE and POST) and the two testing conditions (sitting and walking). We set the significance level at *p*<0.05 and used Tukey Honestly Significant Difference (THSD) post hoc tests for pair-wise comparisons if the walking condition effect was detected. The effect size for the main effect of the ANOVAs was estimated using partial eta squared (*η*^*2*^, i.e., the proportion of variance in each of the outcome measures explained by the exercise effect or walking condition effect) [24, 25]. Following Cohen and previous studies [24, 26, 27], *η*^*2*^ values were interpreted as: 0.02 “small” effect, 0.13 “medium” effect and 0.26 “large” effect. All statistical analyses were performed in JMP version 13 (SAS institute Inc., Cary, NC, USA).

## Results

**Fig 1:**
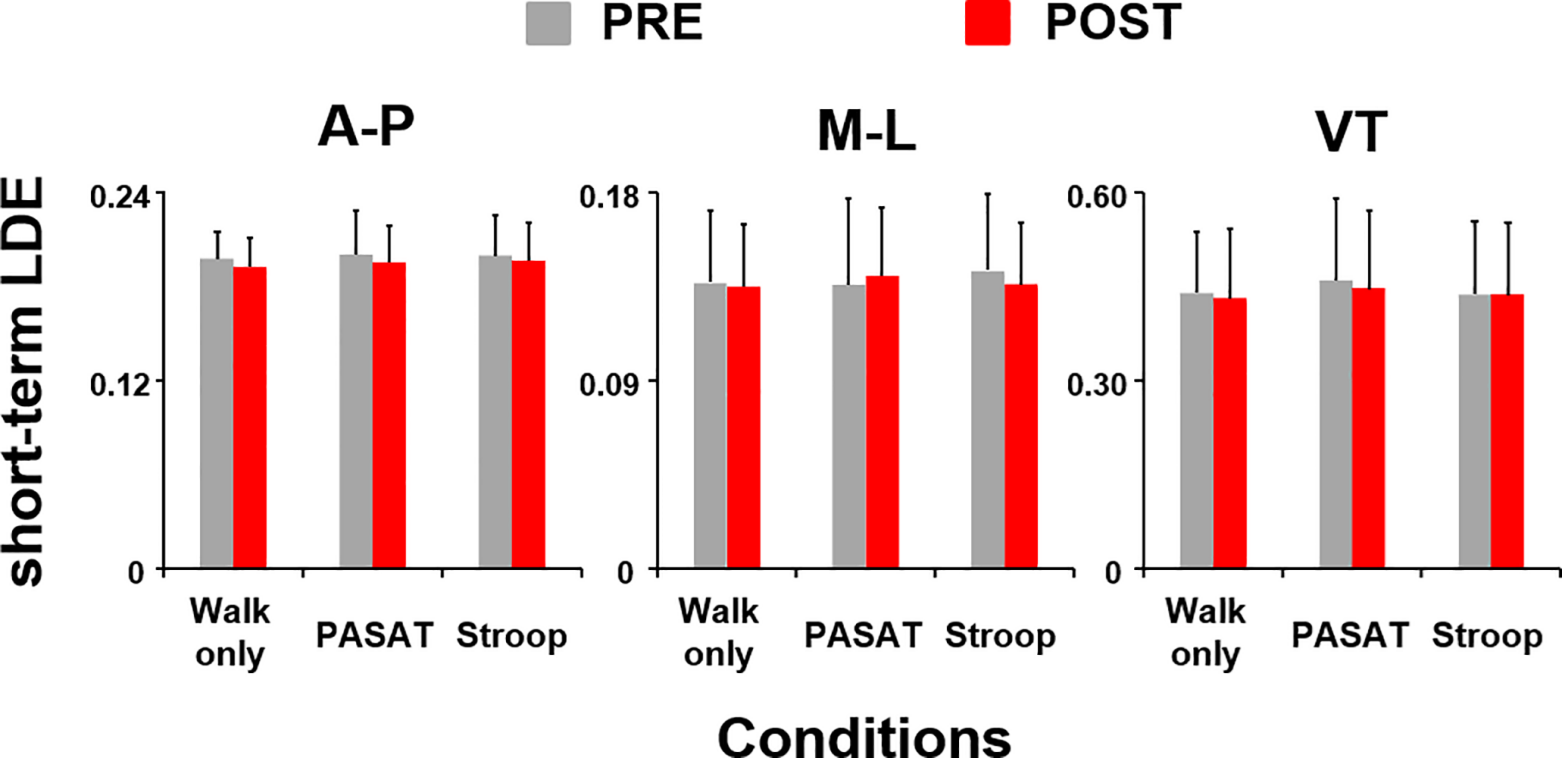
Short-term LDE. Short-term LDE, indicating local stability of the C7 vertebral marker movement, in the anterior-posterior, medio-lateral, and vertical directions before (PRE, grey bars) and after (POST, red bars) the muscle fatiguing exercise session. Error bars represent 1 STD.

Subjects had similar values of short-term local divergence exponents (LDE) for the C7 movements before and after the muscle fatiguing exercise session (A-P: F_(1,85)_ = 1.90, *p* = 0.17; M-L: F_(1,85)_ = 0.04, *p* = 0.84; VT: F_(1,85)_ = 0.57, *p* = 0.45) as well as across different walking conditions (A-P: F_(2,85)_ = 0.43, *p* = 0.65; M-L: F_(2,85)_ = 0.67, *p* = 0.51; VT: F_(2,85)_ = 0.23, *p* = 0.80) (Fig 1). These results indicate that local stability was not affected by the fatiguing exercise or dual-task conditions.

**Fig 2:**
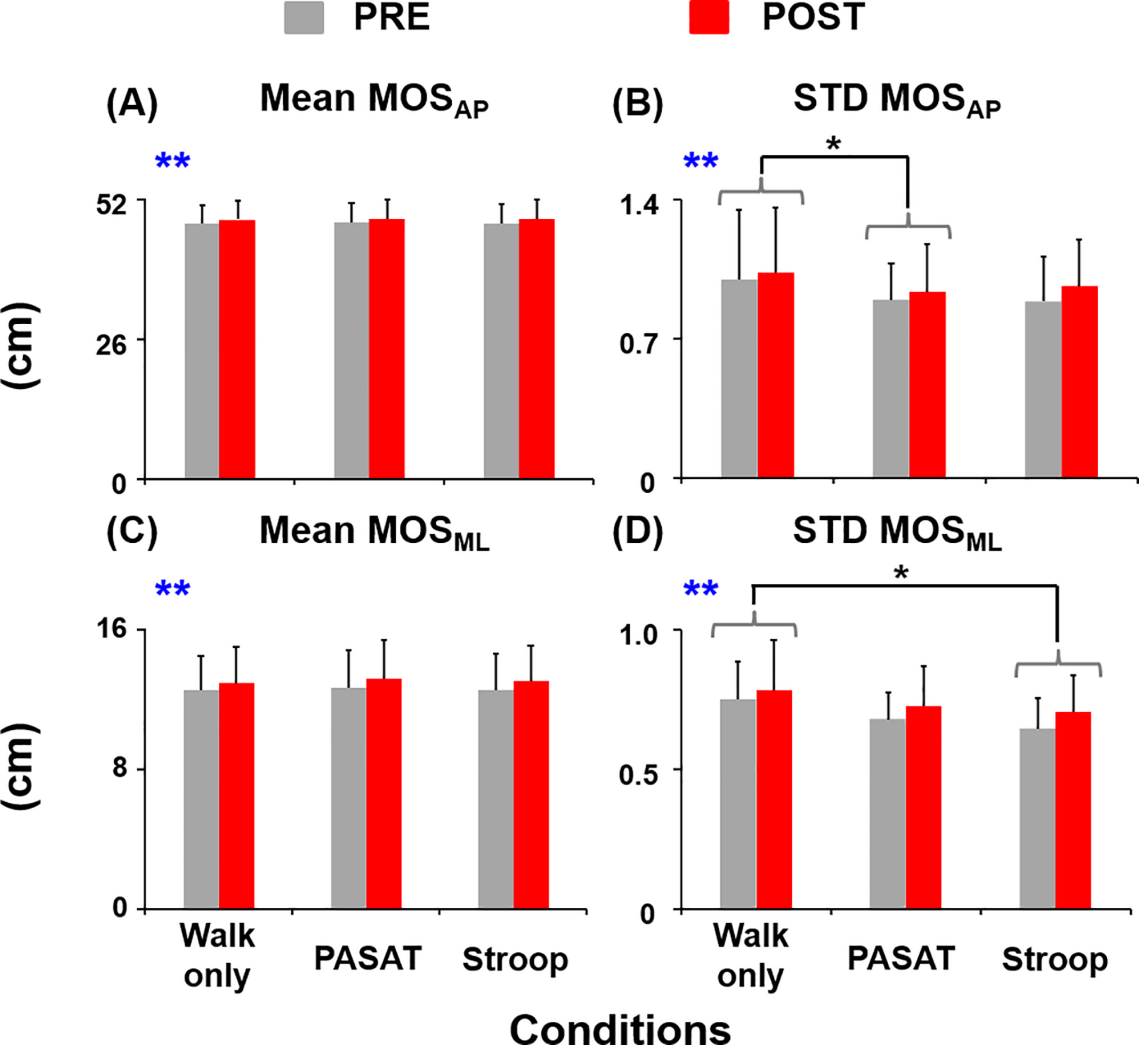
Margins of stability. (A) Mean and (B) variability of MOS_AP_, and (C) mean and (D) variability of MOS_ML_ before (PRE, grey bars) and after (POST, red bars) the exercise session. Error bars represent 1 STD. ** indicates significant difference between the PRE and POST. * indicates significant difference from the walking only condition (THSD, *p*<0.05).

Subjects walked with significantly greater average MOS_AP_ (F_(1,85)_ = 34.38, *η*^*2*^ = 0.29, *p*<0.01) and MOS_ML_ (F_(1,85)_ = 46.24, *η*^*2*^ = 0.35, *p*<0.01) as well as greater variability in MOS_AP_ (F_(1,85)_ = 11.49, *η*^*2*^ = 0.12, *p*<0.01) and MOS_ML_ (F_(1,85)_ = 14.28, *η*^*2*^ = 0.14, *p*<0.01) after the fatiguing exercise (POST) compared to the baseline (PRE) (Fig 2). There were no walking condition effects for average MOS_AP_ (F_(2,85)_ = 0.35, *p* = 0.71) or MOS_ML_ (F_(2,85)_ = 1.57, *p* = 0.21). Subjects walked with similar average MOS_AP_ and MOS_ML_ during the dual-task walking conditions compared to walking only. However, the variability of MOS_AP_ was significantly smaller during PASAT compared to walking only (THSD, *p*<0.05). In addition, the variability of MOS_ML_ was significantly smaller during Stroop compared to walking only (THSD, *p*<0.05).

**Fig 3:**
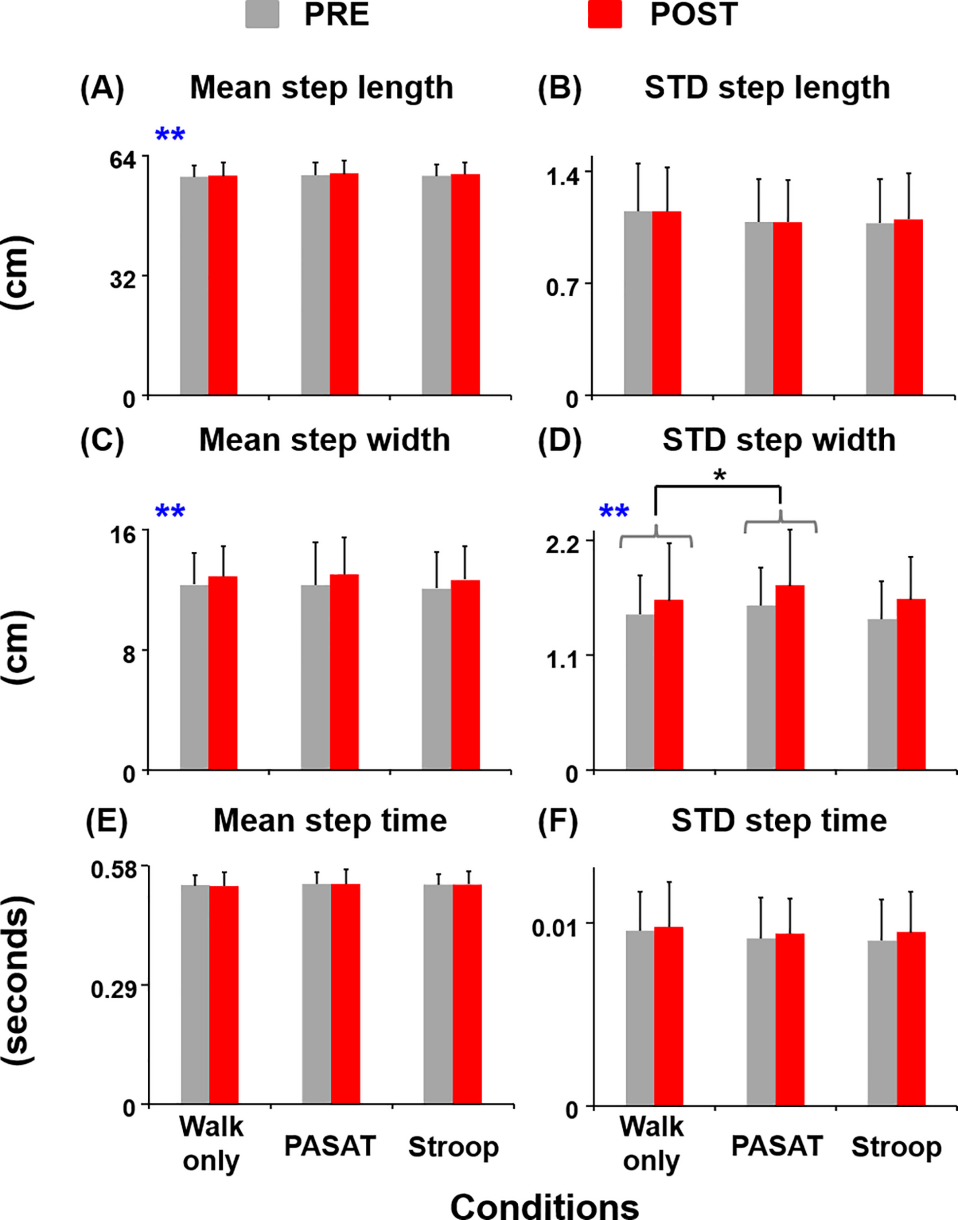
Step measures. (A) Mean and (B) variability of step length, (C) mean and (D) variability of step width, and (E) mean and (F) variability of step time before (PRE, grey bars) and after (POST, red bars) the exercise session. Error bars represent 1 STD. ** indicates significant difference between the PRE and POST. * indicates significant difference from the walking only condition (THSD, *p*<0.05).

Subjects walked with significantly greater average step lengths (F_(1,85)_ = 10.65, *η*^*2*^ = 0.11, *p*<0.01) and widths (F_(1,85)_ = 20.44, *η*^*2*^ = 0.19, *p*<0.01) post exercise compared to PRE (Fig 3). In addition, subjects walked with significantly greater step width variability (F_(1,85)_ = 19.12, *η*^*2*^ = 0.18, *p*<0.01) and showed a trend of walking with greater step time variability (F_(1,85)_ = 2.81, *p* = 0.10) during POST than during PRE. For the walking condition effect, subjects had significantly greater step width variability during PASAT compared to walking only (THSD, *p*<0.05). There was no exercise or walking condition effect for the step length variability or average step time.

**Fig 4:**
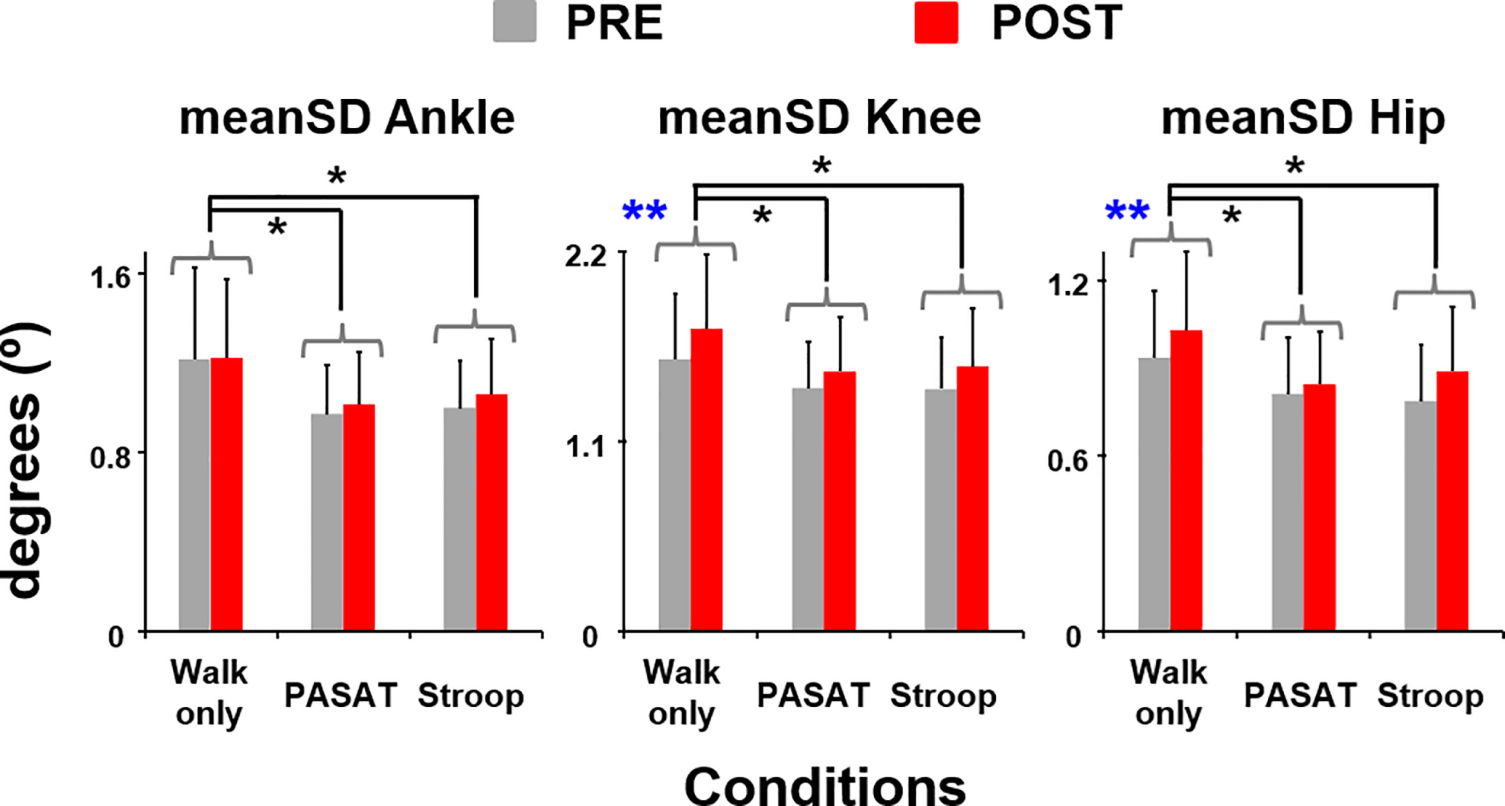
Mean standard deviation (meanSD) of the lower-limb joint motions. meanSD of ankle, knee, and hip joint angles before (PRE, grey bars) and after (POST, red bars) the exercise session. Error bars represent 1 STD. ** indicates significant difference between the PRE and POST. * indicates significant difference from the walking only condition (THSD, *p*<0.05).

Subjects walked with significantly greater knee (F_(1,85)_ = 34.20, *η*^*2*^ = 0.29, *p*<0.01) and hip joint angle variability (F_(1,85)_ = 21.13, *η*^*2*^ = 0.20, *p*<0.01) and showed a trend of walking with greater ankle joint angle variability (F_(1,85)_ = 2.79, *p* = 0.10) during POST compared to the baseline (Fig 4). However, subjects walking with significantly less ankle, knee and hip joint angle variability during PASAT and Stroop compared to walking only (THSD, all *p*<0.05).

**Fig 5:**
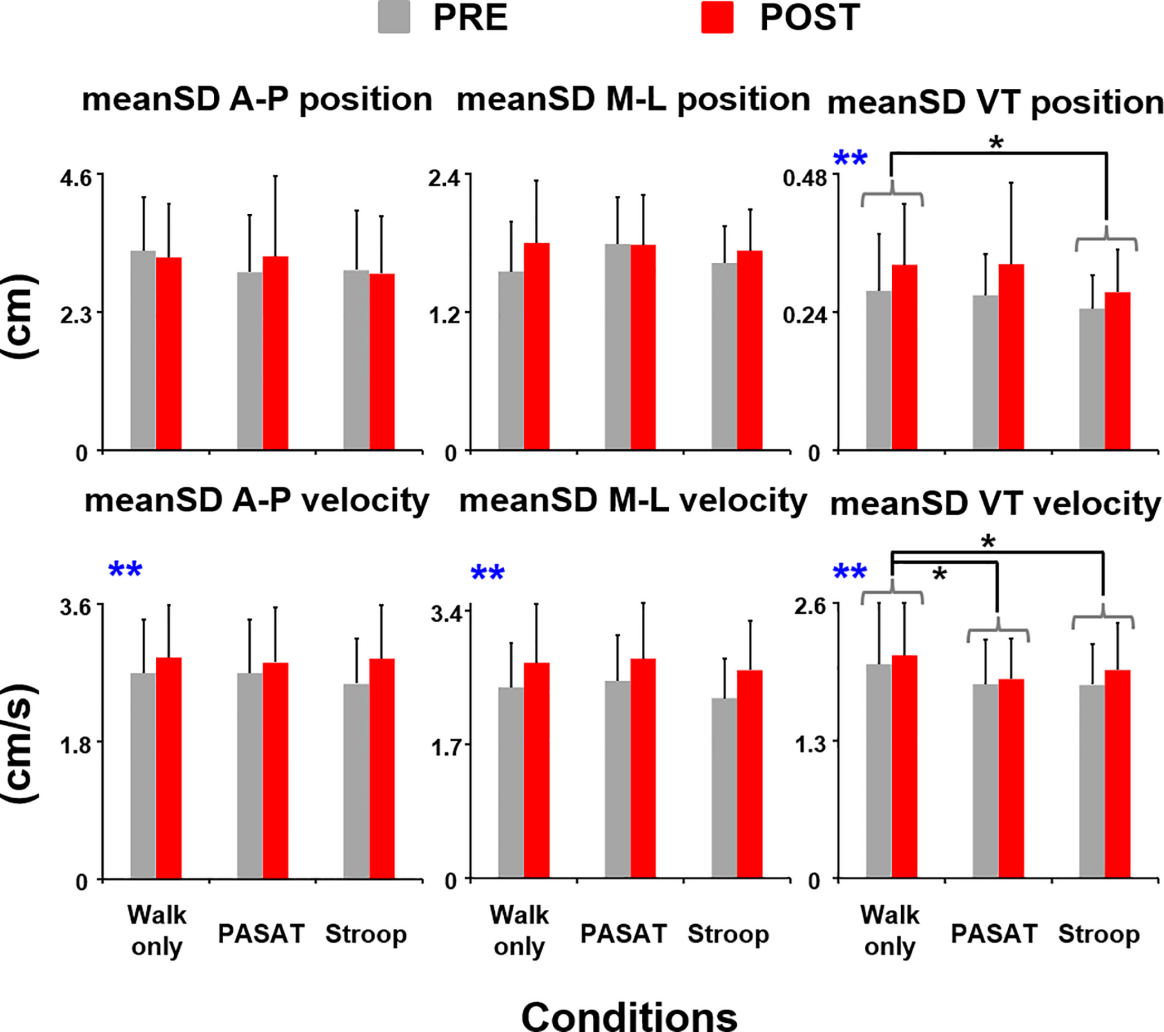
Mean standard deviation (meanSD) of C7 marker positions and velocities. meanSD of C7 marker positions (top panel) and meanSD of C7 marker velocities (bottom panel) in the A-P, M-L, and VT directions before (PRE, grey bars) and after (POST, red bars) the exercise session. Error bars represent 1 STD. ** indicates significant difference between the PRE and POST. * indicates significant difference from the walking only condition (THSD, *p*<0.05).

Subjects showed a trend of having slightly greater lateral trunk position variability (F_(1,85)_ = 2.72, *p* = 0.10) and significantly greater vertical trunk position variability (F_(1,85)_ = 12.14, *η*^*2*^ = 0.12, *p*<0.01) during POST compared to the baseline (Fig 5). However, subjects walked with significantly less vertical trunk position variability during Stroop compared to walking only (THSD, *p*<0.05). There was no walking condition effect for the trunk position variability in the anterior-posterior or mediolateral direction. Subjects also had significantly greater trunk movement variability in all directions during POST compared to PRE (A-P: F_(1,85)_ = 30.32, *η*^*2*^ = 0.26, *p*<0.01; M-L: F_(1,85)_ = 60.77, *η*^*2*^ = 0.42, *p*<0.01; VT: F_(1,85)_ = 11.83, *η*^*2*^ = 0.12, *p*< 0.01). For the walking condition effect, however, subjects walked with significantly less trunk movement variability in the vertical direction during PASAT and Stroop compared to walking only (THSD, both *p*<0.05). There was no walking condition effect for the trunk movement variability in the anterior-posterior or mediolateral direction.

For the cognitive performance, there was no exercise effect or testing condition effect for the two tests (Table 1). Subjects had similar number of errors for the PASAT or the modified Stroop test during sitting and walking as well as before and after the exercise (Table 1).

**Table 1:** Descriptive statistics for the number of errors in the two cognitive tests.

	PRE		POST		Exercise			Condition		
	sit	walk	sit	walk	F_(1,51)_	*η*^*2*^	*p*	F_(1,51)_	*η*^*2*^	*p*
PASAT	23.1±14.8	24.2±13.3	22.4±14.0	20.9±13.7	1.68	0.03	0.20	0.19	< 0.01	0.67
Stroop	3.7±2.2	3.9±3.1	3.9±3.7	3.9±3.5	0.28	< 0.01	0.60	0.14	< 0.01	0.71

For all the dependent variables (i.e., walking and cognitive measures), there were no significant interaction effects (Exercise X Condition). Overall, outcome measures that render significant exercise or walking condition effect mostly yielded “medium” or “medium to large” effect size for the exercise effect and yielded “small to medium” effect size for the walking condition effect except the joint angle variability measures that yielded “medium to large” effect size for the walking condition effect.

## Discussion

Our findings only partially support our hypothesis that healthy subjects would show reduced walking stability, increased gait variability and more errors in the two cognitive tasks after motor fatigue. We found that subjects walked with a similar level of local stability but increased gait variability after the fatiguing exercise compared to the baseline. These results are consistent to the previous findings of Arvin et al (2015) [2] that examined the effects of unilateral hip abductor muscle fatigue. Arvin et al (2015) demonstrated that gait stability, in terms of local stability, was not affected by the fatigue even though subjects had reduced hip position sense and increased gait variability following the unilateral hip fatiguing exercise [2]. We also found that subjects walked with greater margins of stability while having greater variability in their joint kinematics, step measurements, margins of stability (MOS), and trunk movement after motor fatigue. The results indicate that subjects may try to adjust their steps by walking with wider and longer steps and greater margins of stability so they could maintain a similar level of local stability after motor fatigue. Since motor fatigue would affect subjects’ precision in motor control (e.g., greater neuromuscular noise when producing force) [28], increasing MOS by simply increasing step length and width would be a quick strategy to compensate for the effects of increased movement variability on gait stability. Previous studies that investigated the effects of motor fatigue in repetitive manual tasks (e.g., sawing) also showed similar adaptation following motor fatigue [29, 30]. They found that subjects had increased movement variability and altered kinematic patterns but exhibited similar level of stability, suggesting that the changes in kinematic patterns following motor fatigue might help subjects to maintain desired level of stability [29, 30]. Moreover, we found that subjects had similar cognitive performance during single- or dual-task conditions before and after the motor fatiguing exercise. These results suggest that motor fatigue does not affect cognitive performance but walking performance in healthy younger adults.

In contrast to the adjustments made during fatigued gait, we found that subjects walked with similar margins of stability while substantially reducing the variability in their gait and joint kinematic parameters during the dual-task conditions, consistent to the previous findings [17, 31]. These results indicate that a conservative walking strategy was used when simultaneously performing a cognitively challenging task. In the dual-task conditions, the lower stride-to-stride movement variability, indicating higher movement regularity, suggests that there may be less cognitive control involved for the walking component because the focus of attention has been shifted to the cognitive task [32, 33]. Although younger adults may possess the reserve capacity for enhancing the automaticity of walking while prioritizing the cognitive task [33], the “mindless”, inflexible walking pattern may not allow them to maintain sufficient walking stability when responding to unexpected perturbations or under more complex walking environment. We also found that there was no interaction effect of motor fatigue and cognitive task on the walking measures, indicating that adding a cognitive task did not modify the effects of motor fatigue on gait or motor fatigue did not alter the effects of cognitive task on gait. These findings suggest that healthy young adults use distinct adaptive strategies when walking with motor fatigue versus during dual-task conditions by manipulating MOS and gait variability, respectively. It is possible that each of the strategies could help reduce the likelihood of experiencing a very small or negative MOS during walking, which may help maintain local stability to some extent.

Contrary to our findings, Lorist et al (2002) [10] demonstrated a significant interaction effect by showing greater post-fatigue force production variability while performing a cognitive task as well as a greater decline in cognitive performance while performing fatiguing muscle contractions. In Lorist et al (2002) [10], the motor task was a finger force-matching task via the visual feedback and the cognitive task was an auditory choice reaction test that required subjects to provide answers by pressing one of the two response buttons using the other hand. However, the experimental setup of Lorist et al (2002) [10] simultaneously challenged both visual and auditory attention as well as introduced motor-motor dual-task effect that might potentially cause bimanual interference, which is more complex than simply posing a cognitive-motor interference as we tested here. Thus, the nature and complexity of the dual-tasks may affect how motor fatigue interacts with the added cognitive task and its effect on dual-tasking performance.

One major limitation of this study is that we did not include the effects of whole-body motor fatigue but focused on the effects of localized motor fatigue, which could underestimate the effects of motor fatigue on cognitive and/or cognitive-motor dual-task performance. The whole-body motor fatigue is often induced using exercises such as running, walking, ironman triathlon, etc. These whole-body activities could disturb more sensory systems such as visual and vestibular systems as well as generate greater damage to the plantar cutaneous mechano receptors than the localized muscular exercises [34]. Another limitation is that the walking environment used in the study, even terrain and normal walking speed, is not complex, which may not pose a sufficient challenge to the subjects during the dual-task conditions. These two factors might help explain the limited relevant changes found in the current study such as the small changes in the outcome measures due to fatigue and/or dual-tasking and the “small to medium” effect size for the walking condition effect [35]. Future studies should incorporate a more complex walking environment (e.g., uneven terrain, with mechanical perturbations, faster walking speed) to better examine the cognitive-motor interference during walking.

## Conclusions

The current study investigated the effects of motor fatigue on dual-task walking performance in healthy younger adults. Our results demonstrate that subjects use different adaptive strategies when walking with motor fatigue and during dual-task conditions. Nevertheless, subjects adjusted their steps or controlled joint kinematics to maintain similar level of local stability during motor fatigue or dual-task conditions. Our results also demonstrate that motor fatigue did not affect the cognitive performance but motor performance in younger adults. Future studies should introduce both whole-body and localized motor fatigue as well as incorporate a more complex walking testing environment.

## Supporting information

S1 Table Descriptive statistics for walking stability and variability parameters. (DOCX)
