# Supplementary material for: Effects of motor fatigue on walking stability and variability during concurrent cognitive challenges
Source: PLoS One. 2018 Jul 26;13(7):e0201433. doi: 10.1371/journal.pone.0201433 (PMC6062111; doi:10.1371/journal.pone.0201433)
Supplement: S1 Table — (DOCX) [file pone.0201433.s001.docx]

**S1 Table:** **Descriptive statistics for walking stability and variability parameters**

|  | PRE | | | POST | | | Exercise | | | Condition | | | Post-hoc comparison |
| --- | --- | --- | --- | --- | --- | --- | --- | --- | --- | --- | --- | --- | --- |
|  | Walk only | PASAT | Stroop | Walk only | PASAT | Stroop | F_(1,85)_ | *η^2^* | *p* | F_(2,85)_ | *η^2^* | *p* | (vs. walk only) |
| A-P  local stability | 0.198  ±  0.018 | 0.200  ±  0.028 | 0.200  ±  0.026 | 0.193  ±  0.019 | 0.196  ±  0.024 | 0.197  ±  0.025 | 1.90 | 0.02 | 0.17 | 0.43 | 0.01 | 0.65 | n.s. |
| M-L  local stability | 0.137  ±  0.034 | 0.136  ±  0.041 | 0.143  ±  0.036 | 0.135  ±  0.030 | 0.140  ±  0.033 | 0.136  ±  0.030 | 0.04 | < 0.01 | 0.84 | 0.67 | < 0.01 | 0.51 | n.s. |
| VT  local stability | 0.440  ±  0.097 | 0.460  ±  0.131 | 0.438  ±  0.116 | 0.432  ±  0.112 | 0.448  ±  0.125 | 0.438  ±  0.115 | 0.57 | < 0.01 | 0.45 | 0.23 | < 0.01 | 0.80 | n.s. |
| Mean  MOS_AP_  (cm) | 47.6  ±  3.5 | 47.8  ±  3.7 | 47.6  ±  3.6 | 48.3  ±  3.4 | 48.5  ±  3.5 | 48.5  ±  3.4 | 34.38 | 0.29 | < 0.01 | 0.35 | < 0.01 | 0.71 | n.s. |
| STD  MOS_AP_  (cm) | 1.00  ±  0.35 | 0.90  ±  0.18 | 0.89  ±  0.23 | 1.03  ±  0.33 | 0.94  ±  0.24 | 0.96  ±  0.23 | 11.49 | 0.12 | < 0.01 | 3.36 | 0.07 | 0.04 | PASAT |
| Mean  MOS_ML_  (cm) | 12.5  ±  2.0 | 12.6  ±  2.2 | 12.5  ±  2.1 | 12.9  ±  2.1 | 13.2  ±  2.2 | 13.0  ±  2.0 | 46.24 | 0.35 | < 0.01 | 1.57 | 0.04 | 0.21 | n.s. |
| STD  MOS_ML_  (cm) | 0.75  ±  0.14 | 0.68  ±  0.10 | 0.64  ±  0.11 | 0.78  ±  0.18 | 0.73  ±  0.14 | 0.71  ±  0.13 | 14.28 | 0.14 | < 0.01 | 5.83 | 0.12 | < 0.01 | Stroop |
| Mean  step length  (cm) | 58.3  ±  3.0 | 58.7  ±  3.4 | 58.5  ±  3.1 | 58.6  ±  3.5 | 59.1  ±  3.6 | 59.0  ±  3.3 | 10.65 | 0.11 | < 0.01 | 2.84 | 0.06 | 0.06 | n.s. |
| STD  step length  (cm) | 1.15  ±  0.30 | 1.08  ±  0.27 | 1.08  ±  0.27 | 1.15  ±  0.27 | 1.09  ±  0.26 | 1.10  ±  0.29 | 0.97 | 0.01 | 0.33 | 1.69 | 0.04 | 0.19 | n.s. |
| Mean  step width  (cm) | 12.3  ±  2.1 | 12.3  ±  2.8 | 12.1  ±  2.4 | 12.9  ±  2.0 | 13.0  ±  2.5 | 12.7  ±  2.3 | 20.44 | 0.19 | < 0.01 | 0.99 | 0.02 | 0.38 | n.s. |
| STD  step width  (cm) | 1.49  ±  0.37 | 1.57  ±  0.37 | 1.45  ±  0.36 | 1.63  ±  0.54 | 1.77  ±  0.53 | 1.64  ±  0.41 | 19.12 | 0.18 | < 0.01 | 3.87 | 0.08 | 0.02 | PASAT |
| Mean  step time  (sec) | 0.532  ±  0.025 | 0.535  ±  0.028 | 0.533  ±  0.026 | 0.531  ±  0.029 | 0.536  ±  0.030 | 0.534  ±  0.027 | 0.21 | < 0.01 | 0.65 | 2.48 | 0.06 | 0.09 | n.s. |
| STD  step time  (sec) | 0.010  ±  0.002 | 0.009  ±  0.002 | 0.009  ±  0.002 | 0.010  ±  0.002 | 0.009  ±  0.002 | 0.009  ±  0.002 | 2.81 | 0.03 | 0.10 | 0.70 | 0.02 | 0.50 | n.s. |
| meanSD Ankle  (º) | 1.22  ±  0.41 | 0.97  ±  0.22 | 1.00  ±  0.21 | 1.22  ±  0.35 | 1.01  ±  0.23 | 1.06  ±  0.25 | 2.79 | 0.03 | 0.10 | 8.96 | 0.17 | < 0.01 | PASAT  Stroop |
| meanSD Knee  (º) | 1.58  ±  0.37 | 1.41  ±  0.26 | 1.40  ±  0.30 | 1.75  ±  0.43 | 1.50  ±  0.31 | 1.53  ±  0.34 | 34.20 | 0.29 | <0.01 | 17.82 | 0.30 | < 0.01 | PASAT  Stroop |
| meanSD  Hip  (º) | 0.93  ±  0.23 | 0.81  ±  0.19 | 0.79  ±  0.19 | 1.03  ±  0.27 | 0.85  ±  0.18 | 0.89  ±  0.22 | 21.13 | 0.20 | < 0.01 | 17.58 | 0.29 | < 0.01 | PASAT  Stroop |
| meanSD  A-P position  (cm) | 3.32  ±  0.90 | 2.96  ±  0.96 | 2.99  ±  1.00 | 3.21  ±  0.89 | 3.23  ±  1.33 | 2.94  ±  0.96 | 0.04 | < 0.01 | 0.85 | 0.64 | 0.02 | 0.53 | n.s. |
| meanSD  M-L position  (cm) | 1.55  ±  0.43 | 1.79  ±  0.41 | 1.62  ±  0.33 | 1.80  ±  0.55 | 1.78  ±  0.43 | 1.73  ±  0.36 | 2.72 | 0.03 | 0.10 | 0.03 | < 0.01 | 0.97 | n.s. |
| meanSD  VT position  (cm) | 0.28  ±  0.10 | 0.27  ±  0.07 | 0.25  ±  0.06 | 0.32  ±  0.11 | 0.32  ±  0.14 | 0.27  ±  0.07 | 12.14 | 0.12 | < 0.01 | 3.14 | 0.07 | 0.05 | Stroop |
| meanSD  A-P velocity  (cm/s) | 2.70  ±  0.69 | 2.70  ±  0.70 | 2.56  ±  0.59 | 2.91  ±  0.68 | 2.83  ±  0.72 | 2.89  ±  0.69 | 30.32 | 0.26 | < 0.01 | 0.61 | 0.01 | 0.55 | n.s. |
| meanSD  M-L velocity  (cm/s) | 2.42  ±  0.57 | 2.50  ±  0.59 | 2.28  ±  0.50 | 2.74  ±  0.75 | 2.79  ±  0.71 | 2.64  ±  0.63 | 60.77 | 0.42 | < 0.01 | 1.54 | 0.03 | 0.22 | n.s. |
| meanSD  VT velocity  (cm/s) | 2.02  ±  0.57 | 1.84  ±  0.41 | 1.83  ±  0.38 | 2.10  ±  0.50 | 1.88  ±  0.38 | 1.96  ±  0.44 | 11.83 | 0.12 | < 0.01 | 10.06 | 0.19 | < 0.01 | PASAT  Stroop |
